# High frequency root dynamics: sampling and interpretation using replicated robotic minirhizotrons

**DOI:** 10.1093/jxb/erac427

**Published:** 2022-10-23

**Authors:** Richard Nair, Martin Strube, Martin Hertel, Olaf Kolle, Victor Rolo, Mirco Migliavacca

**Affiliations:** Department for Biogeochemical Integration, Max-Planck-Institute for Biogeochemistry, 07745 Jena, Germany; Department of Botany, Trinity College Dublin, Dublin, Ireland; Max-Planck-Institute for Biogeochemistry, 07745 Jena, Germany; Max-Planck-Institute for Biogeochemistry, 07745 Jena, Germany; Max-Planck-Institute for Biogeochemistry, 07745 Jena, Germany; Forest Research Group, INDEHESA, University of Extremadura, 10600, Plasencia, Spain; Department for Biogeochemical Integration, Max-Planck-Institute for Biogeochemistry, 07745 Jena, Germany; European Commission, Joint Research Centre, Ispra, Varese, Italy; Ikerbasque, Spain

**Keywords:** Digital repeat photography, minirhizotron, neural network, root dynamics, root length density, root phenology, root–shoot synchrony, root surface area

## Abstract

Automating dynamic fine root data collection in the field is a longstanding challenge with multiple applications for co-interpretation and synthesis for ecosystem understanding. High frequency root data are only achievable with paired automated sampling and processing. However, automatic minirhizotron (root camera) instruments are still rare and data are often not collected in natural soils or analysed at high temporal resolution. Instruments must also be affordable for replication and robust under variable natural conditions. Here, we show a system built with off-the-shelf parts which samples at sub-daily resolution. We paired this with a neural network to analyse all images collected. We performed two mesocosm studies and two field trials alongside ancillary data collection (soil CO_2_ efflux, temperature, and moisture content, and ‘PhenoCam’-derived above-ground dynamics). We produce robust and replicated daily time series of root dynamics under all conditions. Temporal root changes were a stronger driver than absolute biomass on soil CO_2_ efflux in the mesocosm. Proximal sensed above-ground dynamics and below-ground dynamics from minirhizotron data were not synchronized. Root properties extracted were sensitive to soil moisture and occasionally to time of day (potentially relating to soil moisture). This may only affect high frequency imagery and should be considered in interpreting such data.

## Teaser

Automatic collection of root dynamics data, transferrable between field settings, is described. Images are collected by robotics and processed by machine learning to produce time series comparable with above-ground digital repeat photography.

## Introduction

Plant phenology (seasonal patterns of recurrent events such as leaf growth and senescence) drives interannual variability of the terrestrial carbon (C) sink (Raupach *et al.*, 2011). It responds to environmental conditions such as climate and weather (Richardson *et al.*, 2013), can differ above and below ground (Adair *et al.*, 2019), and is partially determined by life history (Steinaker *et al.*, 2010). While overall growth is also driven by whole-plant resource budgets, root and shoot phenology are not always linked (Abramoff and Finzi, 2015). Above- and below-ground environments differ (e.g. Blume-Werry *et al.*, 2017) and plants temporally partition resource uptake and demand, resource assignment, and activity of individual organs (Herrmann *et al.*, 2016), which may cause this desynchrony.

Tools to remotely monitor ecosystem dynamics above ground are well developed, for instance ‘PhenoCams’ (Luo *et al.*, 2018; Richardson *et al.*, 2018) or satellite-derived vegetation indexes (Wu *et al.*, 2017). However, using above-ground measurements to proxy for below-ground activity is an unreliable assumption (Kutschera, 1960; Abramoff and Finzi, 2015; Ma *et al.*, 2021). Root phenology measurements are needed in natural field contexts, and high frequency datasets are rare (Radville *et al.*, 2016). This is because of the demanding, destructive, non-repeatable nature of traditional sampling. Data scarcity contributes to basic simulation of root dynamics by models, often relying on poorly calibrated root:shoot ratios, simple environmental triggers, or optimality concepts (De Kauwe *et al.*, 2014; Walker *et al.*, 2015). These are hard to validate because high frequency datasets do not exist at field sites providing other data (e.g. C fluxes) for model fitting.

Roots are also crucial for C cycling because alongside microbes, they control decomposition, and are the main C source to soil organic matter (Dijkstra *et al.*, 2021). Thus, misrepresentation of root dynamics affects prediction of ecosystem capacity to sequester carbon. Unfortunately, mesocosm experiments are prone to artefacts which disproportionately affect roots (Poorter *et al.*, 2012) so the benefits of field experiments (Schindler, 1998) are particularly large for the below-ground parts. In natural field conditions, repeatable root observations are made with minirhizotrons (buried observatories and camera systems). Many other advances in root phenotyping (e.g. Nagel *et al.*, 2012; Le Marié *et al.*, 2014; Liu *et al.*, 2021) are only currently possible in more controlled conditions. While there are constraints and biases to minirhizotron usage, they allow non-destructive, repeatable sampling, unlike other methods with other advantages (Maeght *et al.*, 2013; Freschet *et al.*, 2021). Minirhizotron automation has been possible for 15 years (Allen *et al.*, 2007) but still often uses infrequent imaging and manual-driven analysis (e.g. Defrenne *et al.*, 2021). Robotic minirhizotrons for monitoring below-ground phenology of roots could offer many opportunities, but have barely advanced beyond pioneering experiments using these systems (Vargas and Allen, 2008; Iversen *et al.*, 2011; Allen and Kitajima, 2013, 2014).

Automated observations introduce a bottleneck: processing high frequency imagery to calculate proxies of visually identifiable root properties. A manual approach will inevitably not annotate images as fast as collection. Recently, convolutional neural networks (CNNs) show promising results to identify roots in a variety of settings (Rahmanzadeh and Shojaedini, 2016; Vincent *et al.*, 2017; Huo and Cheng, 2019; Alonso-Crespo *et al.*, 2022; Bauer *et al.*, 2022). In particular, field soil minirhizotron imagery has been analysed several times (Wang *et al.*, 2019; Gillert *et al.*, 2021; Han *et al.*, 2021; Bauer *et al.*, 2022; Peters *et al.*, 2022, Preprint; Smith *et al.*, 2022), but transferability between sites and out of agricultural soils is difficult to assess without widespread adoption in new settings. These have also never been applied to high frequency studies where variability between images (e.g. soil moisture and soil animals) may cause instability in the time series data produced.

We built a robotic minirhizotron system (henceforth, RMR) with a per instrument parts budget of €2000, allowing replication. We paired this with an established CNN method (Rootpainter, Smith *et al.*, 2022) previously used in low sampling frequency studies or soil cores (Han *et al.*, 2021; Alonso-Crespo *et al.*, 2022). We processed segmented images via scripts to extract basic root properties (root length density and root surface area from segmentation) at high time resolution.

Besides the image acquisition itself, there are several challenges to reliable and useful long-term field operation. We split these into four objectives which we considered essential to demonstrate the RMR method. These were as follows: (O1) the RMR method can generate architectural traits reliably validated against state-of-the-art computer-assisted manual methods; (O2) the time series generated are consistent between replicated RMRs; (O3) RMRs plus autonomous segmentation and trait extraction produce good quality and stable time series for long periods (under the constraints of this study, several months) in a variety of sometimes adverse field conditions which could otherwise be avoided in manual campaigns (e.g. condensation); and (O4) given the potential imperfection in the first three objectives, these time series are interpretable in relation to system functioning. In this case, we tested if root and leaf development were synchronized through paired above-ground proximal remote sensing, and how well root development could predict soil CO_2_ evolution compared with other commonly measured properties (above-ground greenness, soil temperature, and soil moisture).

It is also important to show method robustness across different study systems. In this study, we performed four separate trial experiments of the RMR method. Two of these were in a mesocosm and two were in field conditions. Each trial experiment tested different combinations of the objectives as summarized in Table 1, and had minor modifications to the instrument design, which are further detailed in the Materials and methods.

**Table 1.** Summary of experimental trails presented in this study

Experiment	Description	Revisions	O1: validation	O2: replication consistency	*n*	Duration (months)	O3: field operation	O4: co-interpretation
E1	Proof of concept (greenhouse)	Camera: IDS 1005XS-C mains power	✓	n/a	1	3	n/a	✓
E2	Cross-instrument consistency (greenhouse)	Camera: DFK AFU050-L34 mains power	✓	✓	8	2	n/a	✓
E3	Field trial (Extremadura, Spain)	Camera: DFK AFU050-L34 solar power	✓	Partial	3^a^	4	Partial	✓
E4	Field trial (Thuringia, Germany)	Camera: DFK AFU050-L34 solar power	✓	Partial	2	2	✓	n/a

We had four objectives: O1, consistency against manual methods; O2, consistency of interpreted data between replicated instruments; O3, operation in real-world field conditions; and O4, co-interpretation potential with other data. ✓ indicates full addressing of objectives. Otherwise partial addressing of O2 through lower replication and O3 via iterative design improvements is indicated. Also shown are instrument revisions, number of viable instruments (*n*) and duration, of the trial

^a^ In E3, we ran eight instruments in the field, but had problems with long-term timekeeping due to low temperatures which limited this number to three concurrent instruments with good data. This issue was subsequently fixed for E4 but the instruments were split between two sites (second site not shown) to limit vulnerability to travel disruption.

The mesocosm experiments were: (E1) using a single RMR in a greenhouse paired with proximal remote-sensed above-ground dynamics and soil CO_2_ fluxes (O1 and O4), and (E2) eight replicated RMRs in a greenhouse paired with proximal remote-sensed above-ground dynamics only (O1, O2, and O4).

The field experiments were: (E3) for 4 months in the autumn in a Mediterranean tree–grass ecosystem paired with ‘Phenocam’ greenness (O1, O3, and O4), and (E4) for 2 months in winter/spring in a temperate grassland (O1 and O3). Field experiments encountered heterogenous soil appearances, condensation, soil animals, root litter, and other disturbances potentially affecting instrument operation, human annotation, and consistency of CNN segmentation across a high frequency time series. Hence we expected a worse validation (O1) for these experiments. Both of these were also replicated but at a lower scale than E2 (O2).

## Materials and methods

### Minirhizotron system design

**Fig. 1.**
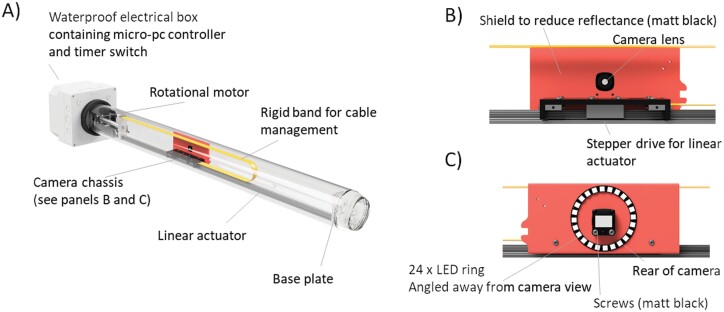
Conceptual diagram of the RMR. The full system (A) moves a camera chassis (B: camera side/C: reverse side) longitudinally and rotationally to image soil in close contact with the tube. The camera chassis is designed to minimize reflectance by mounting the light source on the opposite side of the shield, away from the focal area. In this figure, the light shield and screws are shown in false colours. In the finished instrument, they are matt black to reduce reflectance.

We based our minirhizotron instrument (RMR, Fig. 1) on a movable camera design able to travel in two axes (along a minirhizotron tube, and rotationally around a minirhizotron tube) by using both a rotational motor and a linear actuator (a rotational motor converted into linear motion). All components of the system were purchased unmodified, with the exception of a customized fish-eye lens to allow the camera to focus at ~3 cm within the observatory and the linear actuator, which was built to our dimensions. The RMR uses an (internal) 10/9.6 (external/internal)×100 cm observatory. This is comparable with the only other automatic minirhizotron systems used in published experiments (Iversen *et al.*, 2011; Svane *et al.*, 2019). It takes ~40 min to sample 112 separate images covering the entire tube. Timing of cycles is set by a timer switch and the instrument completes one sampling cycle whenever it is powered on. Robotics are controlled by a Lattepanda development board computer with an integrated Arduino-based co-processor (ATmega32u4, LattePanda, Shanghai, China) which allowed us to control the robotic components with Arduino and the image capture by shell scripts simulating manual operation of the Graphical User Interface (GUI)-based camera software. The light source is provided by a ring of LEDs which were angled away from the image subject to reduce reflectance and only switched on during image capture. The instrument framework was made of black plastic, and all exposed screw heads were painted black for the same purpose. Cable management was achieved by affixing cables to a rigid band only able to bend in the lateral direction. A power supply converter from 12 V to 5 V was used for the PC, camera, and LED ring lights. One sampling cycle required ~10 Wh of power.

RMRs operate at any angle; we ran the RMR horizontally in the greenhouse (E1 and E2) or at an angle in the field (E3 and E4). We tested the RMR across a range of temperatures from –20 °C to ~35 °C (see details later). Images (25 μm pixel^−1^, ~1000 dpi, 6.5 × 4.9 cm in our final system) are captured with a small overlap in .jpg format. These were saved onto removeable 128 GB SD cards; in order to obtain the images, the SD cards were swapped with blank alternatives when the instruments were not powered. A log file was amended at the end of each imaging cycle which could be checked much faster than individual images and easily accessed over WiFi. A single image was <1 Mb, therefore the RMR could sample ~1100 cycles without the SD card being changed.

Over the course of the four experiments, we made some minor modifications to the RMR design as we discovered ways to ensure long-term reliability. In brief, these were: using the best camera possible to ensure good quality images—the most basic step of minirhizotron imaging—in our off-the-shelf design we were limited to a USB-2 connection due to a smaller/more flexible cable than the USB-3 standard. Our optimal camera in E1 (IDS 1005XS-C, IDS imaging Development Systems, Obersulm Germany) was out of stock until a new model in the line by E4, so we used our second choice in E2 and E3. In E4, we further added a magnetic rotational sensor to allow a more reliable return to ‘reset’ position between cycles and limit rare but critical mechanical failure, and a GPS clock to prevent a drift in the internal BIOS clock which we noticed occurred unpredictably at low temperatures. This problem rendered images uninterpretable in time series context. Although this modification was successful in E4 we further tested this robustness in a –20 °C cold room. These latter modifications were not known to be necessary in early instrument design but were critical for long-term, reliable field operation (O3). Further information about the design can be found in Supplementary Protocol S1, and a summary of the hardware and duration differences between experiments in Table 1.

### Greenhouse experiments

We designed two greenhouse experiments: (E1) with one RMR (128 880 individual images) and (E2) with eight RMRs (216 000 individual images). In E1 we sampled every 2 h and had additional ancillary instruments; in E2 we sampled every 6 h without paired ancillary instruments. Each mesocosm [140 cm (L)×40 cm (W)×30 cm (H)] was filled with soil sieved to 0.5 cm (roots and stones removed), harvested from Meusebach, Germany. Soil C:N was 18:2. We did not measure soil density (our soil was highly artificial) but did not expect this to differ between mesocosms in E2, set up at the same time. Soil covered the upmost surface of the RMR by 2 cm, so the field of view (FOV) spanned 2 cm to 12 cm below the soil surface.

Each RMR observatory was located in a mesocosm unit, extending internally 90 cm from one end. As here the RMR was horizontal, the first ~10 cm of the length, usually partially above ground, was not sampled. Aside from the camera, which we changed because of a supply issue (resulting in worse image quality in E2), and minor modifications for cable management, the RMR design was the same. In E1 we used a ‘fresh’ observatory installed factory clean, but in E2 we re-used an experimental set-up for manual measurements which we cleaned before starting. Thus, this latter experiment potentially had artefacts on the tube surface, which we discuss later. Both experiments were run in the greenhouse of the Max Planck Institute for Biogeochemistry, Jena, Germany.

In both E1 and E2, a three species seed mix (80/15/5% by mass *Anthoxanthum odoratum*, *Plantago lanceolata*, and *Medicago scutellata*) was scattered evenly over the soil surface, kept moist to germinate. Additionally, in both E1 and E2, water was provided at irregular intervals (3–10 d) and varying volumes (between 2 litres and 6 litres per mesocosm), evenly over the mesocosm surface via a watering can with a distributor nozzle to mimic field rain events. Partway through each experiment, we then withheld water to move the system towards a state of drought-induced root senescence. This senescence period was longer in E2 (1 month) than in E1 (2 weeks).

All mesocosm units in both greenhouse experiments were included in the FOV of a standard ‘PhenoCam’ set up (Sonnentag *et al.*, 2012; Richardson *et al.*, 2018), modified for indoor use via a fish-eye lens. The camera settings were defined from the ‘PhenoCam’ protocol (Richardson *et al.*, 2018). Images were collected between 12.00 h and 13.00 h local time. We defined a single region of interest (ROI) for each entire mesocosm unit, parallel to the long axis of the minirhizotron. Data were processed as in field-scale studies (e.g. Luo *et al.*, 2018, 2020). During this time, artificial lights were switched off and watering never took place during this hour. FOVs during each experiment were stable and images were available from the entire period. From the images, we obtained the daily green chromatic coordinate (GCC), the ratio between digital numbers in the green channel and the sum of the digital numbers in red, green, and blue (RGB) channels, commonly used to represent canopy greenness in field studies. GCC has been found to outperform the normalized difference vegetation index (NDVI) in forests when assessing vegetation cover and condition and supressing scene illumination variation (Nijland *et al.*, 2014). Using greenness indexes assumes that healthy vegetation is greener than less healthy vegetation.

We also installed soil moisture and soil temperature probes [a combination of EC-5 soil moisture probes (Li-COR Biosciences, Lincoln, NE, USA) and ML-3 soil temperature probes (Decagon Instruments, USA)], and measured root biomass through six 4.5 cm×13 cm ingrowth cores in each mesocosm installed at initiation. These were evenly spaced, at 5 cm from the wall of the unit and an equal distance from the central minirhizotron tube. These were retrieved and root biomass measured by sieving the soil to 2 mm and weighing the washed and dried (60 °C, 3 d) roots. In E1, we sampled six unreplicated time points (to minimize disturbance and because of the limited area of soil not occupied by the minirhizotron or the gas exchange measurement), plus the start (no roots). In E2 we sampled the start (no roots), three unreplicated time points, and then one time point at the end of the experiment with three replicates per mesocosm.

Finally, in E1, we measured system gas exchange using a Li-8100A Infrared Gas Analyzer (IRGA), a Li-8100-104 opaque long-term chamber (Li-COR Biosciences) every half-hour. We let vegetation grow within the chambers. CO_2_ concentrations were converted to fluxes using the R package RespChamberProc; in general, the fit of all observations was very good (>99% with an *R*^2^ of 0.99). Occasional, unplanned periods of power disruption in both experiments prevented data collection and image capture but did not affect the subsequent minirhizotron images.

### Field trials

The third (E3, 68 432 images, 203 cycles per instrument) and fourth (E4, 53 760 images, 240 cycles per instrument) trials were in two different field settings. E3 was at Majadas de Tiétar (Spain), a Mediterranean wood–pasture (El-Madany *et al.*, 2018; Nair *et al.*, 2019). We deployed eight RMRs as in E2 powered by per-instrument solar panels coupled with an external 12 V battery and charge controller. We sampled twice daily from October 2019 until January 2020 (~22 800 images per instrument). Majadas de Tiétar is a Mediterranean ecosystem where the growing season lasts from autumn until late spring, but much undecomposed root litter remains after dry summer (Nair *et al.*, 2019). The soil is an Abruptic Luvisol with a sandy upper layer and a thick clay layer starting at 20–40 cm. In E3, we encountered an unpredictable issue with the BIOS clock when night temperatures fell below 0 °C, affecting accurate timekeeping (and hence reference time for images collected) at all subsequent sampling points. Therefore, we show summary data from two instruments where timekeeping was not disrupted across all 4 months and a third composite instrument made from two instruments with partial time series (i.e. only ‘true’ time-referenced data used, with three instruments over 80% of the time series, gapfilled via linear interpolation for periods missing data). We also use a unified meteorological dataset for a large-scale ecosystem experiment (‘MANIP’) at the site. Air temperature was the mean of three measurements made at 2 m height at three eddy covariance towers located within ~950 m of each other. Precipitation was likewise the mean of precipitation recorded at each tower. GCC used in this experiment was the mean of ‘pasture’ GCC captured from a ‘PhenoCam’ digital camera used for site comparison between the three towers (Luo *et al.*, 2020) with a 3 d average taken for a daily value.

E4 was conducted in plots of the Jena biodiversity Experiment (Roscher *et al.*, 2004; Weisser *et al.*, 2017) in Jena, Germany from 1 February to 4 April 2022 where mean air temperature was 4.5 °C and minimum temperature was –7.5 °C. Here two instruments ran from mains power sampling four times daily. A single meteorological station was located in the centre of the experiment site. Air temperature was measured at 2 m and rainfall from a single precipitation gauge. This site is a loamy Eutric Fluvisol in the floodplain of the river Saale.

In E3 and E4, observatories were installed at 40°, E3 in May 2015 and E4 in August 2020. In this manuscript we show root properties averaged over the whole observatory depth, which in both E3 and E4 reached 45 cm underground. A summary of objectives and hardware differences between E1 to E4 is given in Table 1.

### Processing minirhizotron imagery for root trait time series

**Fig. 2.**
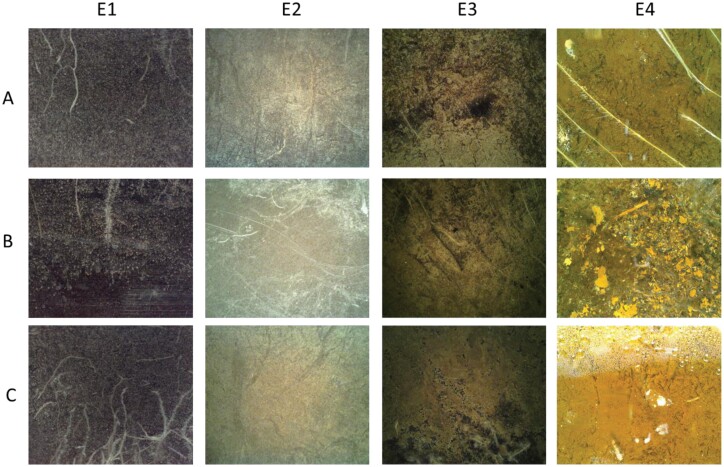
Example of three random images (A–C) from each of our experiments. Each image is originally 2292 × 1944 pixels. The soil appearance and image quality were very different between experiments due to a combination of different image sensors and different image subjects. Notable examples of segmentation challenges are scratches and tube surface artefacts (E1B and E2B), litter and remains of dead roots (E3), and condensation and soil animals (E4C).

**Fig. 3.**
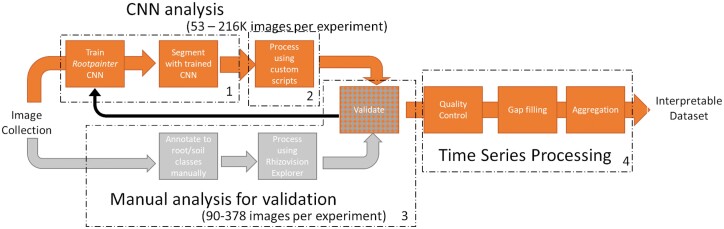
Summary of the image processing workflow from collection to interpretation. Images are collected and processed with the CNN analysis path, with a subset of images being used for validation data using the manual analysis path. Numbered boxes refer to the steps in the text in the ‘Processing minirhizotron imagery for root trait timeseries’ section. (1) CNN training and segmentation, (2) processing segmented binary images, (3) validation, and (4) post-processing for time series. If validation was poor in Step 3, we continued to train the CNN.

Images collected from all four experiments were very different and contained different artefacts (Fig. 2). However, we processed all images in the same way, over four steps. These were: (1) segmentation of the images into binary maps using a trained CNN; (2) processing of the binary images to extract root properties; and (3) validation of these properties against manual annotation (to fulfil O1). Once we were confident in the validation, we (4) applied quality control checks, gapfilled and aggregated the resulting data to produce time series which could be compared with each other across instruments (O2), and interpreted over long periods (O3) and with other data (O4). This overall process is summarized in Fig. 3.

In Step 1, we trained a CNN with a GUI, allowing corrective annotation (Rootpainter, Smith *et al.*, 2022). For this study, we hosted our own remote server, using local computer cluster GPU cores. We used a random model trained from scratch in each experiment. We aimed for separation of RMR image pixels into two classes—roots and soil—annotating the totality of each root. Living and dead roots were separated based on connectiveness, colour, and expert user opinion. We paid attention to closely growing roots but allowed their annotations to touch when there was no clear separation in the image. We started without any pre-annotated images, training on 308 (E1), 300 (E2), 400 (E3), or 350 (E4) complete 2292 × 1944 random images. This had the benefit of allowing the annotator to see the context of the whole image although there are advantages in loading time and training time to work on subimages, as recommended in the original publication. In all cases, we had stopped training when we (qualitatively) assessed that the segmentation was not improving, the model had undergone 60 epochs training without improving fit, and the corrective annotation had processed at least 300 images. Training data are a major issue for automatic minirhizotron studies which have only short intervals between images but may have sudden changes (e.g. changes in soil colour following rain). Random annotation is unlikely to capture these events, and targeting these times introduces bias. Because of the camera change (Table 1), and potentially because of the legacy of previous roots around the observatory, images in E2 and E3 were also challenging to both human annotations and CNN segmentation due to low contrast, poor focus, and faint marks on the tube surface. E3 also had high litter content, leading to ambiguity between roots and litter, and rapid soil appearance changes as the ecosystem was released from summer drought. Thus, high throughput of training data via corrective annotation/active learning strategies (e.g. Beluch *et al.*, 2018; Budd *et al.*, 2021; Ren *et al.*, 2021) offers a major advantage in such unpredictable tasks.

In Step 2, we processed the segmented dataset to produce basic root traits through scripts in Python 3. In this analysis we extracted root length and segmented root surface area (sRSA), which we defined as the total area of roots in the segmented image. To do this, we first applied a filter on the segmentation with a total area of <0.5 mm^2^, which we treated as noise. Because our minirhizotron images contain partial aspects of more than one plant’s root system, root network area was the total area remaining and was not necessarily connected. This is equivalent to ‘Network Area’ in the modern Rhizovision Explorer software tool (Seethepalli *et al.*, 2021); that is, the total pixels in the segmented area but without the network connectivity from a single root system. We divided this by the area of the image for a value as a percentage. We skeletonized the root area of all segmented images to one pixel wide, and calculated the total length by summing the number of pixels in the skeleton. We further converted root length into root length density (RLD) by dividing the total root length by the total area of the image as we were only observing those portions of root which encountered the observatory and not whole roots. RLD is a common property in analyses relating to soil exploration and resource uptake from fine roots (Freschet *et al.*, 2021), while sRSA incorporates variation in diameter and can be assumed to correlate more closely to observable biomass if density is stable. For RLD, we did not adjust for diagonal connections or prune branches of the skeleton, but otherwise this is similar to the simplest level of analysis using Rhizovision Explorer. This software can be directly paired to the Rootpainter CNN and has been successfully used to process imagery from agricultural soils with similar accuracy to manual annotation (Bauer *et al.*, 2022), and can potentially extract many more features of interest. However, our datasets in natural ecosystems were challenging to both the CNN segmentation and for the human operator to choose appropriate settings for the software over tens of thousands of images. We also faced a software constraint from the huge number of segmented images we had to process in one go, restricting the use of GUI-based software. Hence, we used a simpler programmatic option to generate our time series where we had the advantage of frequent resampling to make up for individual inaccuracy.

In Step 3, we use 378/170/140/90 random manually annotated images in E1/E2/E3/E4 as validation data. Images were annotated by multiple users (E1, E2, and E3) or the lead author (E4) with anonymous file names in GIMP software (https://www.gimp.org/). In E1 and E2, we tried to produce high quality annotation with up to an hour of annotation time per image. For E3 and E4 these were just a ‘fast-pass’ annotation (maximum 10 min annotation time per image, often less) deemed suitable for high-throughput time series where a wide range of soil and root conditions were encountered, and covering these was as important as strict pixel-level annotation in a limited number of training images. We compared at image level; that is, we did not assess the fit of individual pixels. By working at the image level, we discarded the advantage the minirhizotrons have in tracking individual roots, because we were interested in the main patterns relatable to phenology rather than pixel-wise accuracy on the tiny fraction of the overall dataset where it would be feasible to produce extremely detailed validation data. We were also using GCC, a coarse image-level index for above-ground time series anyway, and it was easier to produce a validation set without needing full or connective annotation of individual roots or complete accuracy in root diameters.

To process these validation images, we used Rhizovision Explorer (Seethepalli *et al.*, 2021), a state-of-the-art root imagery tool. We processed the manual pixel maps to extract sRSA and RLD without relying on the segmentation or our post-processing scripts for RLD. Thus our validation of the RLD step is the most conservative option, and differences could have arisen either from the trained CNN segmentation mismatch with manual annotation or from the skeletonization mismatch with the software routine. In the analyses which followed, we used RLD for time series whenever possible as this ignored potential inconsistencies in root diameter which would disproportionately affect volume (i.e. sRSA). This could have affected both segmentation and manual mark-up, and been artefactual or real. In the comparison of C efflux in E1 and for growth rates in E1 and E2 we used sRSA. We made this latter choice because sRSA is closer to C pools in biomass than RLD despite potential diameter uncertainty. Statistical comparisons between the validation and CNN-segmented data are described in ‘Statistical analyses’ below. In the event of poor validation, we returned to Step 1 and trained the model further.

Finally, for Step 4, we post-processed the images for interpretable time series. After considering images in E1 and E2, we realized that horizontal installation meant that images on the top of the tube were different from those at the bottom. Thereafter, we treated the top of the tube as ‘truth’ and used this 3/8 of a complete rotational series for further analyses. In E3/4, where the instruments were deployed in a conventional fashion in the field, we used the sides of the instrument (excluding 1/4 of images at on the top and bottom of the tube) for analysis. In all experiments, we then also took a daily average across traits extracted from all valid images for a single value which was not biased by any potential sub-daily cycle as an artefact of the segmentation (Supplementary Fig. S1). For overall time series in the field experiments only, we gapfilled an aggregate time series level accounting for the occasional missing cycle via linear interpolation between successive points. In the greenhouse experiments, including the generalized additive model (GAM) analysis which addressed O4, we used only the non-gapfilled data points. We then took a further 3 d rolling average in common with field above-ground phenology approaches (Migliavacca *et al.*, 2011a; Aasen *et al.*, 2020). To calculate growth rates in E1 and E2, we then took the linear slope in this smoothed three daily average over a 5 d window (we found similar results for a 4, 6, or 7 d window; Supplementary Table S1).

### Statistical analyses

We performed statistical analyses for O1 and O4. For O1, we tested if there was a linear relationship between manual and CNN-segmented root properties. We used reduced major axes regression in the lmodel2 package (Legendre, 2018) in R (R Core Team, 2018). With this analysis, we calculate the determination coefficient and slope of observed (x, annotation) versus estimated (y, CNN sRSA) in the imagery, accounting for a similar magnitude of the error in x and y. We also tested if systematic bias (i.e. slope not equal to 1) between manual and CNN analysis was introduced by comparing the difference between normalized (0–1) CNN and manual properties against confounding variables such as time, soil moisture, and absolute roots in the image and checking for linear trends.

To analyse the effect of mesocosm conditions on CO_2_ efflux in E1, we fit a GAM (Hastie and Tibshirani, 1986; Wood, 2006), implemented via mgcv (Pedersen *et al.*, 2019). This allowed an independent, non-linear smooth to be fit per predictor; for example, an sRSA effect on soil respiration could occur due to more root biomass, other root presence effects such as priming of soil carbon turnover, drying or wetting of soil due to hydraulic lift or transpiration, or root turnover itself. For predictors, we used normalized (0–1) soil moisture content, temperature, 5 d slope of normalized sRSA and normalized GCC, and the mean normalized ‘biomass index’ (mean normalized GCC and normalized sRSA, combined due to high concurvity). We fit a univariate smooth for each without interactions. Otherwise, concurvity in all cases was <0.7. We used the restricted maximum likelihood (REML) method to estimate smooths to reduce overfitting. We compared the variable importance in these models using the varIMP function in the caret package (Kuhn, 2008).

## Results

In this section, we first compare validation against manual tools (O1), then assess O2, O3, and O4 on a per-experiment level across the four experiments.

### Validation against manual tools

**Fig. 4.**
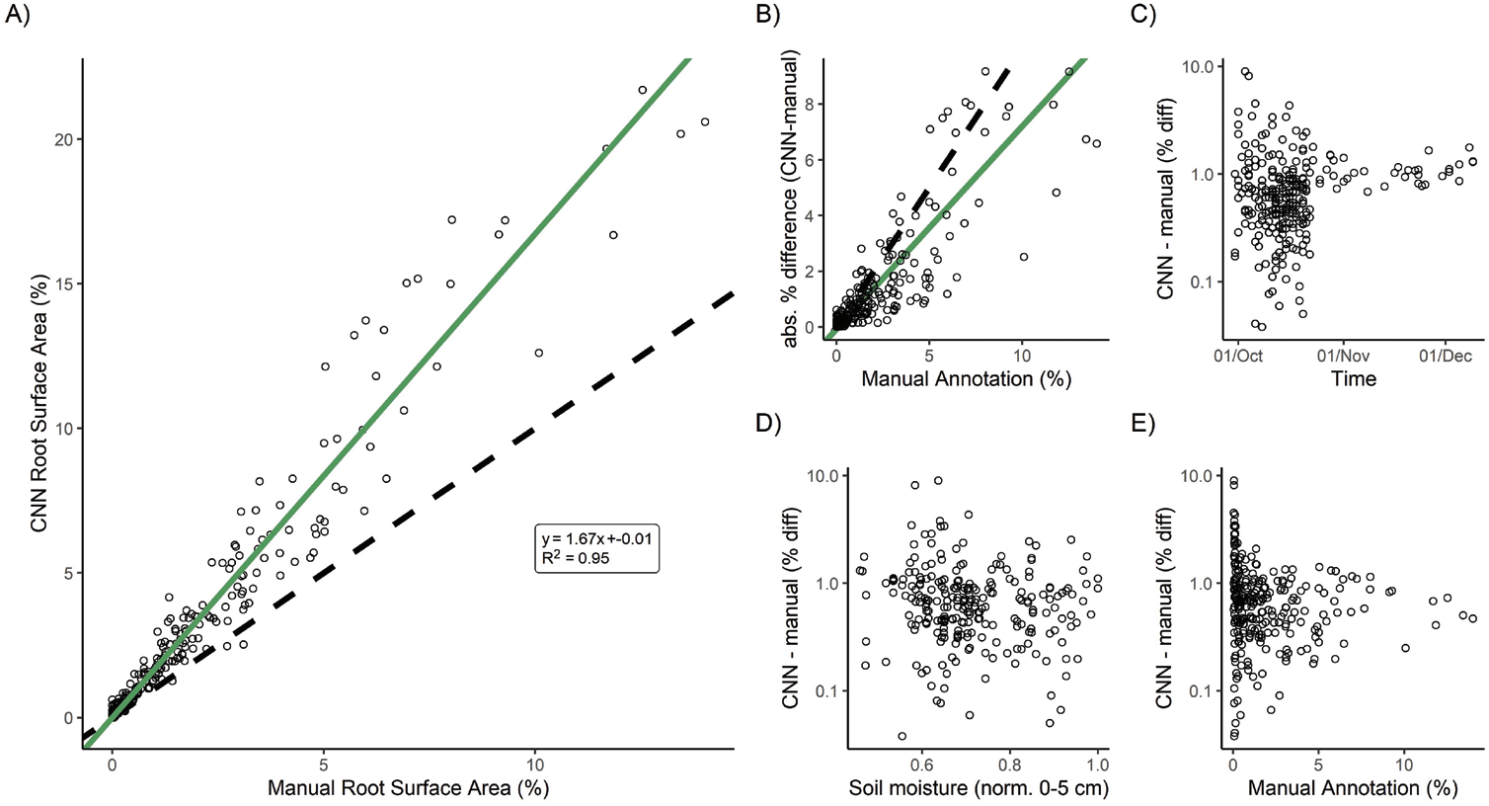
(A) Validation of CNN annotation for segmented root surface area (sRSA) against independent manual root annotation in E1. The green line shows a reduced major axis regression of the two variables, while the dashed line is a 1:1 relationship. Statistics show the equation of significant sRSA fit. (B) Absolute percentage difference between manual sRSA and CNN-classified sRSA, and relative percentage difference standardized to manual annotation over (C) time, (D) soil moisture content in the 0–5 cm soil at the time of sampling, and (E) manual sRSA. The increased absolute error at higher manual sRSA was expected. The clustering of data towards the early part of (C) is due to an uneven validation dataset.

To examine if the RMR method worked in a comparable fashion to state-of-the-art manual methods (O1), we validated sRSA and RLD from our simple extremely high-throughput method against the GUI-based tool ‘Rhizovision Explorer’ for the same properties. Overall, we found a good correlation for sRSA in E1 (*R*^2^=95%, Fig. 4A), E2 (*R*^2^=66%), E3 (*R*^2^=81%), and E4 (*R*^2^=92 %). The relationship between manual and CNN sRSA for E2, E3 and E4 is shown in Supplementary Fig. S2. In the mesocosm experiments, the CNN tended to identify more pixels as roots than did humans (slope >1), while in the field it was the reverse (slope <1). We investigated the drivers of this disagreement in E1. While there was an expected relationship between manual cover and absolute difference between CNN and manual mark-up (larger values could be more wrong, Fig. 4B), there was no effect of time, soil moisture content, or manual cover on the error relative to the manual cover (Fig. 4C–E). Hence, we could trust the segmentation on average over the whole time series in terms of dynamics and, if adjusted by a linear transformation, in terms of magnitude.

**Fig. 5.**
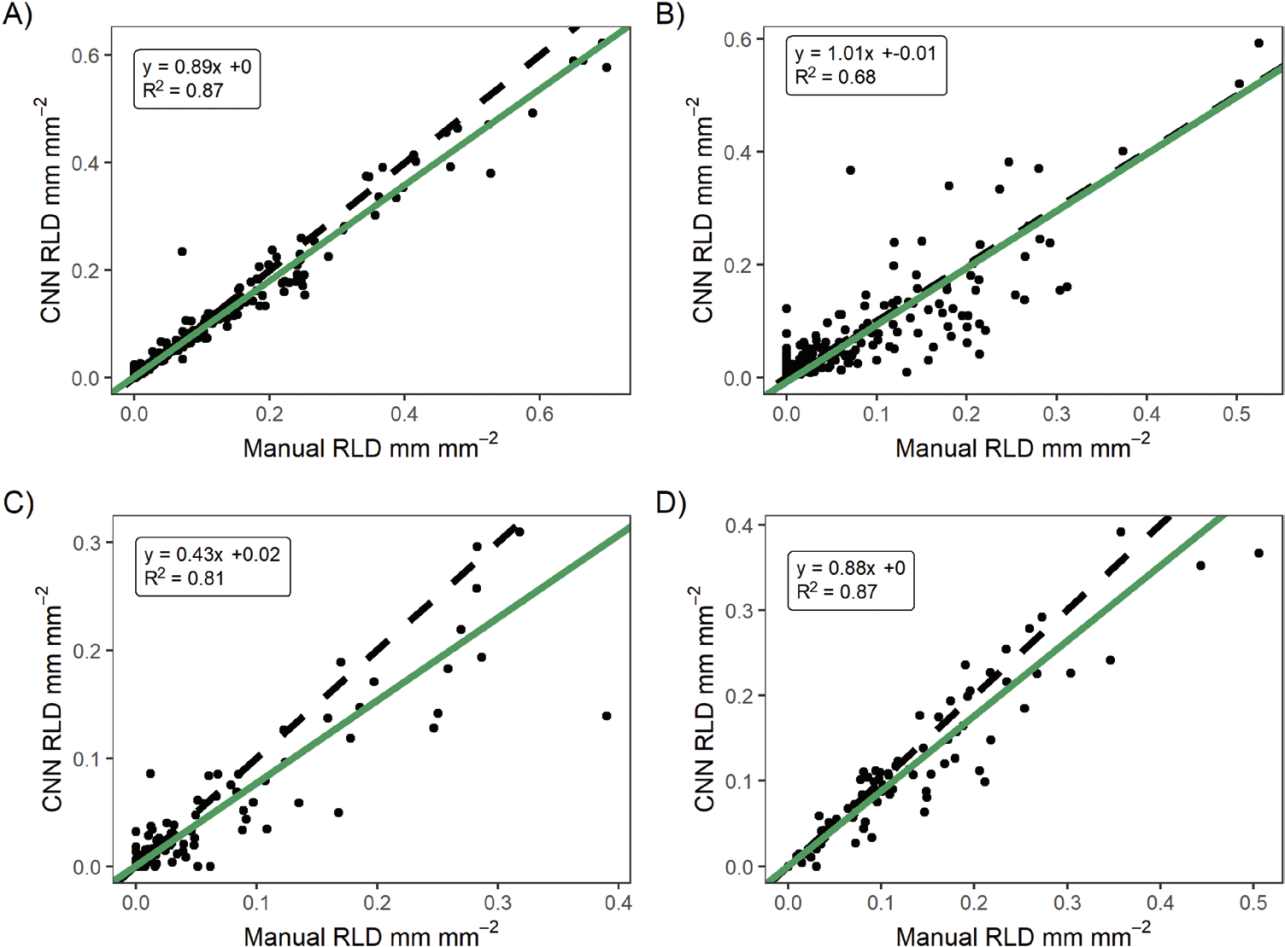
Validation of CNN annotation for root length density (RLD) using our simple script for the very large dataset against extraction of RLD on manual annotated imagery using the Rhizovision Explorer software tool. The green line shows a reduced major axis regression of the two variables, while the dashed line is a 1:1 relationship. (A) is E1, (B) is E2, (C) is E3, and (D) is E4. In general there was good agreement, with disagreement due to both the accuracy of the CNN in segmenting (see Fig. 2) and potentially differences in the RLD extraction routines.

We also found a good validation of RLD. While we used a simpler extraction routine with less tuneable parameters than Rhizovision Explorer, our RLD extraction agreed closely (Fig. 5). The *R*^2^ in E1 was 97%, in E2 was 68%, in E3 was 81%, and in E4 was 87%.

### E1: biological interpretation

**Fig. 6.**
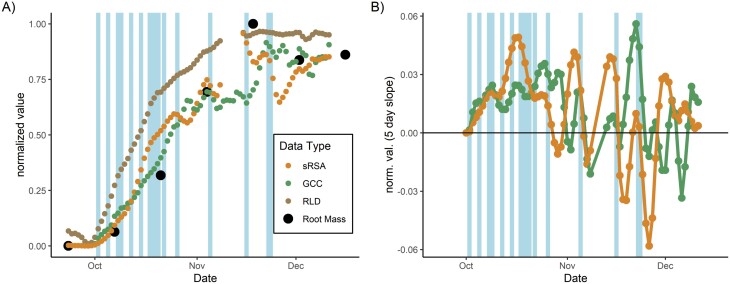
Above–below-ground time series from the mesocosm experiment. (A) The relative, 3 d smoothed segmented root surface area (sRSA), root length density (RLD), GCC (greenness of above-ground vegetation), and root mass, showing parallel development all scaled 0–1. (B) The 5 d slope of biomass-related values (i.e. rate of change) where at some periods GCC is increasing faster than sRSA and at others sRSA is increasing faster than GCC. Blue lines show watering events which are scaled in width relative to the volume of water applied.

In E1 we paired the CNN sRSA and RLD time series with the GCC from PhenoCam imagery and the absolute root mass measurements (Fig. 6A, O4). We found a good correlation between normalized root mass and sRSA (Pearson *r*=0.96), while RLD was smoother and slightly less well correlated (Pearson *r*=0.94). Some of the instability in sRSA followed the last four watering events (Fig. 6A), only effectively possible to observe with the high-throughput CNN processing and not related to general trends in SWC.

We also compared rates of change (‘growth’) of the above- and below-ground indexes conceptually close to biomass. In general, this differed between GCC and sRSA (Fig. 6B) except at the start of the experiment. Some of this variation was due to the short period after watering, but this variation continued when we ceased water inputs. In general, sRSA growth continued to be positive at the end of the experiment even when the GCC was stable or decreasing as the soil slowly dried out.

**Fig. 7.**
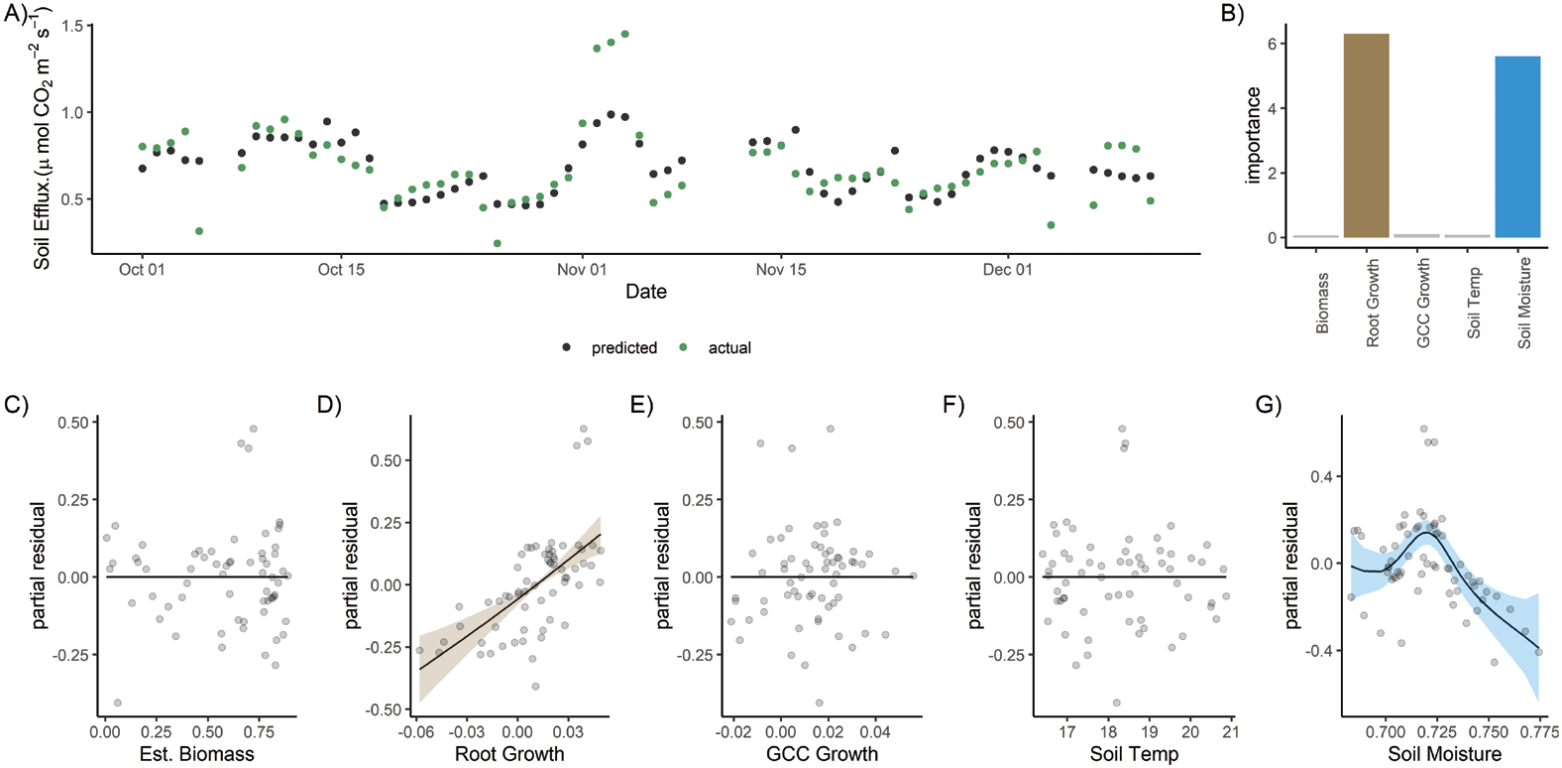
Summary of the GAM model fit for E1. Segmented root surface area (sRSA) correlated with GCC so we used a mean of the two after normalization to represent biomass in the model (Est. Biomass). We were able to predict daily CO_2_ flux fairly well, with an *R*^2^ of 46% (A). The model contained univariate smooths for Est. Biomass, shown versus partial residuals in (C), the 5 d slopes (i.e. growth rate) of sRSA (D), GCC (E), soil temperature (F), and soil moisture content (G). Shaded area shows 2× SE. When variable importance was considered (B), the slope of root cover (i.e. change in amount of roots, birth, or death) was a much better predictor than that of biomass.

Using the GAM on E1, we were able to explain 46% of the total variation in CO_2_ soil efflux with the best-fitted model (Fig. 7A, O4). In this model, soil moisture content (*P*<0.001) and root growth (*P*<0.001) were significant. Root growth was most important in determining soil CO_2_ efflux (Fig. 7B); specifically, increasing root growth rate had a positive effect on soil CO_2_ efflux (Fig. 7D). Soil moisture was also important but had a non-linear shape—the fastest root growth was at intermediate moisture contents and the overall effect of the residual was smaller. In contrast, absolute ‘biomass’ index, and GCC slopes, our proxy for leaf growth rate, did not have an effect (Fig. 7C, E) on soil CO_2_ efflux.

### E2: instrument consistency

**Fig. 8.**
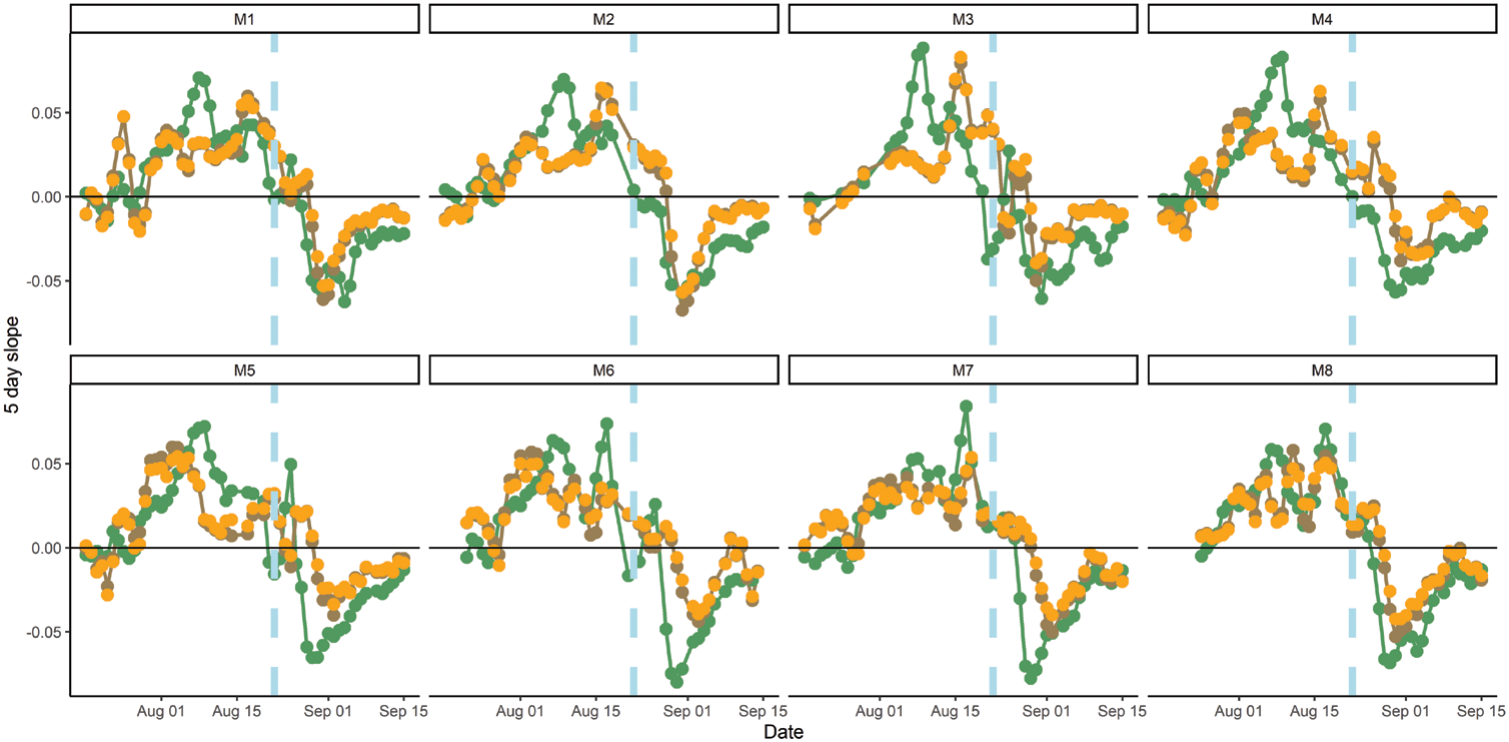
Five day rolling average slope (‘growth rate’): brown is sRSA, orange is RLD, green is GCC across eight mesocosms in E2. RLD and sRSA are closely correlated. The vertical blue line indicates the last watering date, the horizontal black line is 0, and the transition point between positive growth rates (above-) and a decrease in the index (below-) indicating yellowing or disappearance of roots identified by the CNN trained on ‘living’ roots.

Overall, in accordance with O2, a similar time series was extractable from each mesocosm (Supplementary Fig. S3). Correlation between GCC and RLD was between 0.7 and 0.96 (O3). Notably, GCC increased faster than sRSA in the first four mesocosms (Fig. 8; Supplementary Fig. S3). The mesocosms were arranged in numerical order, suggesting that artefacts relating to the position of the mesocosm (e.g. light) within the greenhouse may have driven a difference. Once watering ceased, root growth rate declined less steeply than GCC in all mesocosms and remained positive for longer than GCC growth rate, indicating a continued production of roots even as the above ground began to yellow. The less negative growth rates at the very end of the experiment may indicate that the area segmented as roots could not decrease further.

The roots extracted from ingrowth cores at the start of the experiment matched the overall time series (when normalized between mesocosms, see Supplementary Fig. S3B). However, the last data point, collected at harvest, did not agree, and the measured root biomass was still high when sRSA had dropped.

### E3: Mediterranean/root litter field trial

**Fig. 9.**
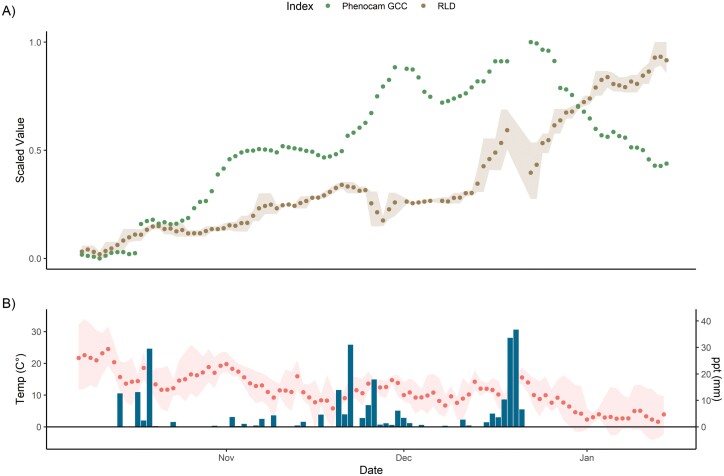
(A) Three day normalized GCC (green chromatic coordinate, green) and mean RLD (brown) from three instruments at the Majadas de Tiétar site. Error bands on root cover correspond to maximum and minimum mean segmented root cover in the aggregation period. (B) Air temperature and precipitation, taken as daily mean, with error bands on temperature corresponding to observed maximum and minimum. The high errors in late November are due to instrument drop-out, reduction to two instruments in this period, and subsequent instrument replacement. A short period (2 d) is missing in mid-December as batteries failed to charge in very cloudy weather. Root growth began later in the Mediterranean growing season than leaf growth and continued even when GCC was declining in midwinter. There was some instability in the root index which may have followed precipitation, although this was not larger than relative instability in GCC.

The first field trial was conducted in a Mediterranean ecosystem so the period studied was early in the growing year. Examining mean total change in RLD over the whole depth and all instruments sampled, we observed the growth period starting in November–December, and after the initial green-up of the above-ground system as detected by GCC (Fig. 9). Certain short-term instability in this index appeared to be related to periods of rainfall (as in E1). We additionally filtered out a short period (19–21 December) where all images across all instruments were anomalously very poorly illuminated (i.e. low current to the LEDs, probably due to poor charging of solar panels in bad weather). In general, temporal variability and noise in RLD were of similar relative magnitude to site-level GCC (O4). RLD generally was lagged following positive GCC change, unlike E1, potentially due to the conventional installation angle here and subsequent better coverage of the whole soil. E3 was halted by the failure of accurate timekeeping across all instruments (i.e. partial failure of O3), which we subsequently addressed in E4.

### E4: Germany/low temperature field trial

**Fig. 10.**
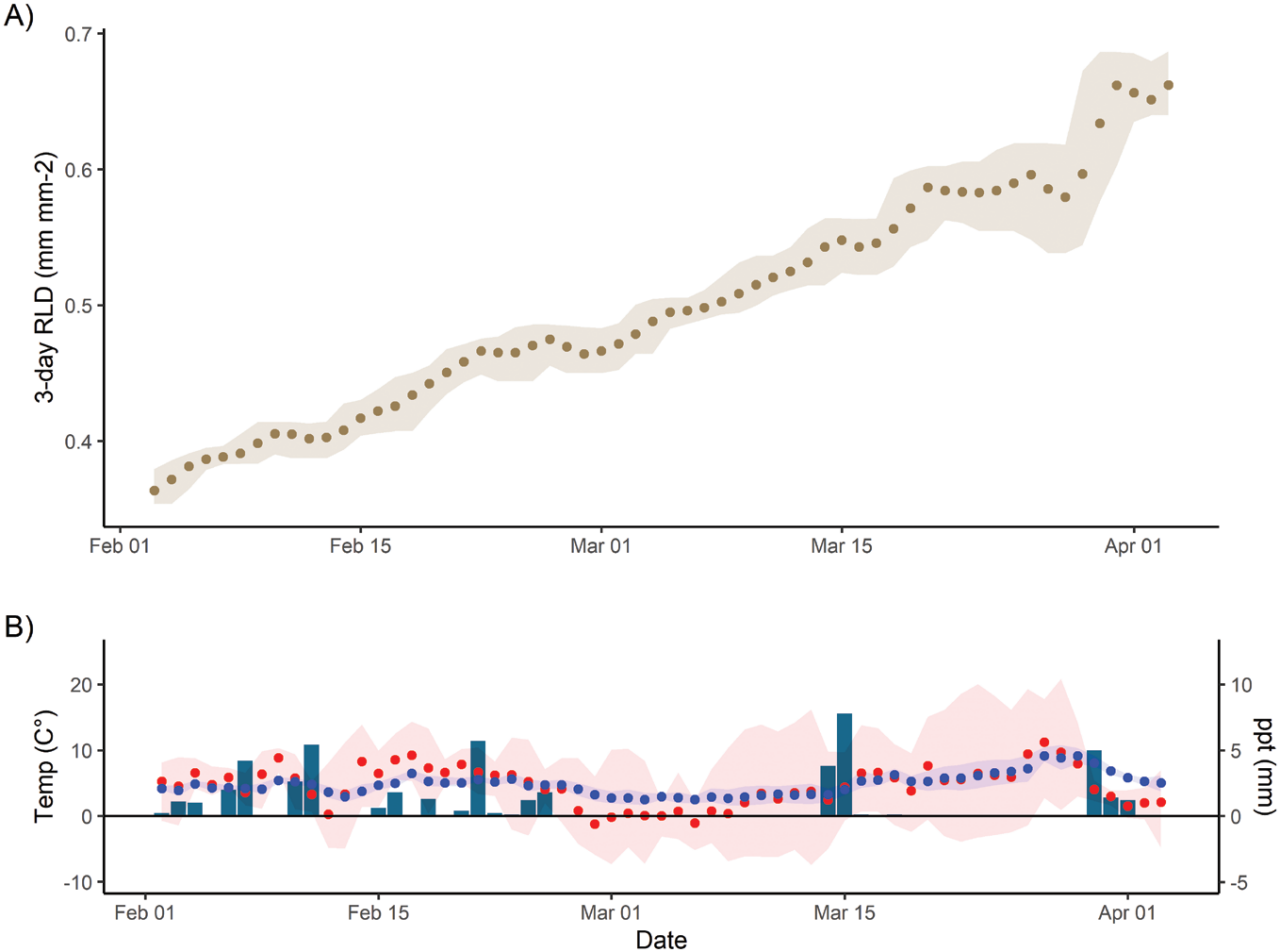
(A) Three day mean normalized root length density (brown) from two instruments at the Jena Experiment site showing growth in February and March. The error band shows the maximum and minimum segmented RLD during the aggregation period. This winter trial shows robustness of the RMR to cold temperatures; the mean air temperature (red) and soil temperature at 8 cm (blue) in (B) are shown with an error band corresponding to maximum and minimum daily temperatures which fell as low as –9 °C. Unlike E3, it did not appear that in this experiment there was a sensitivity in root cover to precipitation.

In E4, instruments ran without issue under winter conditions more severe than those which had previously caused timekeeping failures, fulfilling O3. We observed root growth through February and March (Fig. 10) with periodic increases in RLD and periods of no net growth which did not qualitatively appear to be linked to site conditions. We had occasional unexplained periods where the measurement cycle did not start; this affected ~5% of observational periods with no relationship to temperature or humidity. The images were confounded by condensation and soil animals, which we were largely able to successfully train around, so did not have the same vulnerability to soil moisture and precipitation as E1 and E3. There was no relationship between the root surface area we segmented over the whole 0–40 cm depth sampled and air temperature, precipitation, or soil moisture. Hence the observations can be considered real root growth in this time.

## Discussion

### Use of automatic minirhizotrons

The minirhizotron technique is the best method to measure high frequency root phenology and dynamics in the field (Freschet *et al.*, 2021). Automation of the whole workflow, from image collection to analysis, is essential for high time resolution data. Here, we presented a new automatic system. It is designed for relatively affordable budgets (we used €2000 per instrument including development). We therefore replicate between instruments and can frequently resample through time. After refinement, the method fulfilled all our objectives of (O1) long-term operation, (O2) replication consistency (Fig. 8), (O3) field robustness (Figs 9, 10), and (O4) interpretability with other datasets (Figs 7, 9). Hence, we consider the RMR method as shown here robust for field data collection. We do, however, note that methods such as PhenoCams (Sonnentag *et al.*, 2012; Richardson *et al.*, 2018) or networks for spectral vegetation indices (Gamon *et al.*, 2015) are instrument standardized, but automatic minirhizotrons must combine robotics and imaging across multiple point sensors each with a short FOV. These technical aspects are advancing rapidly. Soils are also complex, and below-ground imaging needs different magnification, sampling time, and power supplies depending on the question. Hence, we recommend against standardization of every aspect of RMR design if time and expertise is available to build to exact demand. In particular, cameras can be tooled to specific applications, as neural networks probably require retraining to new sites and tasks, and images must always be validated against properties of interest from manual annotation. Other optimizations of design could also exclude images based on intended use angle (as we did after image collection for all images), and reduce image storage demands and capture time, although this would also result in incomplete imaging of the entire observatory.

Affordable instruments are particularly necessary as replication is a key shortcoming of many field studies (Filazzola and Cahill, 2021; Yang *et al.*, 2022). These issues are especially acute for minirhizotrons given the short FOW, potential artefacts (Joslin and Wolfe, 1999), and huge range of soil and root appearances. The necessary modifications and COVID-19 led to field trials E3 and E4 not using all instruments from E2, but we observed consistency and reliability in patterns within the instruments used (O2). While we discarded some of the interpreted images, we covered every sampling time point which is, to our knowledge, the numerically largest minirhizotron dataset so far analysed. Data collected at such high frequency have multiple issues even in this simple analysis, with implications for application to more complex situations. The majority of this discussion concerns these issues.

### Interpreting high resolution root image data

Neural network methods are an obvious approach to analysing minirhizotron images, like many other root phenotyping applications (Atkinson *et al.*, 2019). They are also necessary to exploit the high volume of imagery from automated sampling. While we did not develop a new CNN, we applied an established algorithm (Smith *et al.*, 2022) for root images, because of the corrective annotation interface. This method performed well and met O1 (Figs 4, 5; Supplementary Fig. S2) despite the complexity of our images and the lack of time series consideration in neural network design. Interestingly, our own segmentation and trait extractions did not agree perfectly with manual annotation and the state-of-the-art manual tool. Because each process contained two steps (Fig. 2; CNN: training data input+model fitting; manual: manual annotation+Rhizovision Explorer analysis), the consistent differences (overestimation in the field, underestimation in the greenhouse, Supplementary Fig. S3) could have been due either to a bias in the training data (i.e. accuracy in entire root area) or to an effect of the model fitting. The former may have occurred due to the rapid annotation for the manual data and the latter perhaps because the contrast between roots and soil differed between images. The trade-off between throughput and accuracy is particularly important for the large datasets enabled by automatic instruments.

More interpretive root phenotyping (Bauer *et al.*, 2022) can also be paired to the Rootpainter CNN. In theory, many further architectural traits can be extracted with such a workflow. However, application to automatic measurements and field settings relies on high quality imagery, a CNN able to minimize artefacts in unpredictable circumstances, and subjective parameter choice by the analyst; therefore, we did not attempt this here. The large size of the automatic datasets also influenced our choice to process the segmented imagery ourselves and avoid using GUI-based tools. The ability to run complex processing routines transparently from the command line would be a major advance for applying such methods to large automatic datasets and perform sensitivity analyses for tuning parameters in automated trait extraction.

Automatic root identification from automated sampling is demanding on training data but also on validation. We used annotated images produced for other CNN trials for validation data. These were human best practice and not objective truth, so some difference between segmented and annotated images should be expected. Using a CNN meant we could process the data faster—an entire experiment was segmented in 1–3 d once the model was trained. Human annotations could also be outperformed in both consistency and accuracy alongside throughput in identifying true properties of interest by a sufficiently sophisticated neural network well trained on good quality images. However, we expected worse validation under field than greenhouse conditions because of the variety of soil and root appearances in the images. This was generally true, although the camera change for E2 and E3 meant that these validated less well than E1 and E4. (Figs 4, 5; Supplementary Fig. S3). E2 was also negatively affected by previous use of observatories (note the points in Fig. 6B with no manual sRSA but a segmented area), and E3 followed a long fallow period so had many images with a ground truth of no roots contributing to its *R*^2^. These ‘difficult’ images thus had multiple reasons for annotation to disagree with segmentation. In general, a suitable imaging sensor and attention to minimizing observatory artefacts is critical for homemade minirhizotrons. However, above-ground indexes are typically coarse in space and time [e.g. 3 d averaging PhenoCam data (Aasen *et al.*, 2020)], thus similar accuracy to E1 (95% sRSA/97% RLD) or E4 (92%/87%), even E2 (66%/68%) or E3 (81%/81%), with the less powerful camera is sufficient for generating plausible root time series (e.g. Figs 6–8; Supplementary Fig. S5). While in all four cases the CNN segmentation (plus simple processing for RLD) did not perfectly correlate 1:1 with manual sRSA and RLD (Figs 4, 5; Supplementary Fig. S3), we were not validating or training for true pixel-level identification. To minimize these differences rather than use a linear conversion (as we would do in this experiment to interpret from segmented data), we recommend very high levels of effort being put into pixel-perfect annotation of training data. This increases the effort to train for a sufficiently general model. In any case, human root image interpretation is potentially biased by annotator, and other methods such as soil core processing have their own artefacts. Considering that some conversion between measurements will always be necessary, consistent quantitative CNN interpretation has major advantages for throughput for root dynamics.

In the mesocosm E1, we found a good agreement in physical validation between root mass and minirhizotron sRSA (Fig. 6A, *r*=0.96), and also in the growth period of E2 (but not the last, replicated measurement, discussed later). This is despite the potential to lose very fine roots with the common 2 mm sieve method. Validating agreement with biomass data would be an important step for field interpretation. Minirhizotrons are not the best method for absolute biomass estimates at a single time, but further consideration is due to dynamic biomass estimates as the temporal resampling aspect cannot be achieved otherwise. There are differences in properties measurable from minirhizotrons and other methods (e.g. Milchunas, 2009; Addo-Danso *et al.*, 2016; Nair *et al.*, 2019), but minirhizotrons are a reasonable biomass index with appropriate conversions used (e.g. Johnson *et al.*, 2001; Sullivan and Welker, 2005; Brown *et al.*, 2009; Lee *et al.*, 2017). Indeed, root identification relates directly to biological structures around a minirhizotron observatory. This can potentially be paired more easily with density and C contents than leaf greenness of a whole canopy, if the short FOW can be offset by sufficient replication. A key future goal is to ascertain whether well-known artefacts of roots around minirhizotrons (Vamerali *et al.*, 1999; Rytter and Rytter, 2012) can be understood to correct for these issues, and indeed if these artefacts vary with phenology or environmental conditions.

A further issue with our segmentation of high frequency images related to unrealistic variation after rain/watering (Figs 6, 9) and, in E1 and E2, between different times of the day (Supplementary Fig. S2). Like similar issues using classifiers for near infrared-enabled minirhizotron images (Svane *et al.*, 2019), sudden changes in soil colour/reflectance led to erroneous pixel identification and subsequent unrealistic changes in roots identified. We note that in Fig. 8, this instability is not larger than similar short-term patterns in greenness above ground, potentially due to illumination conditions. For the sub-daily artefact, this was not explainable by soil moisture, perhaps because very local condensation at minirhizotron surfaces was not represented in the sensors. Indeed, sub-daily variation only became apparent after roots had colonized the sides of the observatories, suggesting that the CNN was misidentifying pixels in close proximity to roots. Inspection of the images confirms this explanation (Supplementary Fig. S1). Diel variation in root diameter (Huck *et al.*, 1970) or hydraulic redistribution (passive movement of water via roots from wet to dry soil (Ryel, 2004) at night and transpiration during the day drying these areas may provide an explanation which could bias a CNN more than human annotation. If this was a segmentation artefact which affected the immediate ‘rhizosphere’ only, this would not have been detected by our soil moisture sensors.

There are several potential solutions. The first is to aggregate, smooth, or throw out data, which disregards information from high resolution sampling but is common practice in proximal remote sensing at ecosystem level (e.g. using smoothing splines, Migliavacca *et al.*, 2011a). We followed a smoothing strategy in this study, only considering daily means. However, if one wants to analyse sub-daily data with potential diel patterns, this is not a viable solution unless using time series decomposition (e.g. Biriukova *et al.*, 2021). Secondly, one could post-process with human intervention, applying an adjustment or a separately trained model, to periods with problematic changes. This interferes with the image index so is not favourable. Thirdly, one could train on more data, particularly around periods of difficulty. This exacerbates training data issues, especially if problematic events are rare but important, and may bias the model towards fitting these conditions at the expense of a general good fit. Fourthly, consistency in segmentation between sequential images could be used to filter for ‘true’ observations (i.e. basing interpretation on objective priors about root growth). Finally, a wholly different model structure incorporating other factors such as soil moisture or rainfall (i.e. describing when these issues could occur) or time series information [e.g. via recurrent network architectures such as long short-term memory approaches (Hochreiter and Schmidhuber, 1997), multivariate time series classification (Ruiz *et al.*, 2021), or other time series classification (Fawaz *et al.*, 2019)] could be built. This has the advantage of processing data without bias, if training data are selected fairly, at the cost of a more complex model and/or more variables to measure alongside root imagery.

### Field robustness of our techniques

Both automation of measurement and image analysis are more advanced in simple artificial systems than in field measurements, where litter, soil animals, hydrology, and soil appearance complicate imagery. Instruments tested in controlled environments such as E1 and E2 and analysis methods also need to be robust in the field to study phenology. Hence we consider operation under field conditions (O3) a highly important part of our overall study. While E3 was compromised by camera quality and the structurally complex system, we produced a time series plausible from previous work at the site. In this ecosystem, autumn root growth continues through winter, unlike above-ground vegetation indexes in most years (Nair *et al.*, 2019), and continuous operation under winter conditions (low temperatures, poor insolation) is critical to capture dynamics of ‘out of season’ root growth observed in many ecosystems (e.g. Shane *et al.*, 2009; Blume-Werry *et al.*, 2016). The instruments operated consistently on solar power for 4 months with 95% uptime. While we do not show a whole phenological year or include mass root death in the summer drought, this period contained undecomposed root litter following the arid summer, confusing for both human annotators and the CNN. Difficult minirhizotron images are troublesome for even experienced annotators (Peters *et al.*, 2022)— a key advantage of a well trained automated approach is consistency. Our target was also a robust index comparable with above-ground digital repeat photography (e.g. Migliavacca *et al.*, 2011a; Sonnentag *et al.*, 2012) rather than an exact match of segmentation to annotation. A key difference between minirhizotrons and proximal remote sensing is segmenting roots from soil and then interpreting this segmentation rather than a simple image index such as ‘greenness’ on a defined ROI. Because we were segmenting complex features, minor variations in image setting may introduce short-term variation. Future efforts could benchmark acceptable consistency for representativeness of phenology rather than requiring the same accuracy on a high-resolution dataset as in a finer scale analysis from minirhizotrons. On the other hand, neural network approaches could be trained to directly interpret properties such as root length rather than segmenting then interpreting, although this requires alternative models and data-demanding training if multiple traits are of interest.

While we encountered problems under field conditions in E3, we corrected these in E4. The system ran long term successfully after night-time temperatures as low as –7.5 °C without issues. In this relatively ‘easy’ (stone-free and loamy) soil, we were able to achieve our second strongest correlation with manual annotation. We could train around soil animals and condensation in this wet and cold part of the year, and found that roots were growing in this temperate winter. sRSA almost doubled over the 60 d of the trial. Growth did not appear to be related to meteorological conditions, suggesting that (i) these conditions were unlikely to be seriously biasing our CNN segmentation and (ii) this early-season growth may be driven by intrinsic cues and mobilization of stored sugars (e.g. Turner *et al.*, 2007; Maeght *et al.*, 2015) rather than immediate photosynthesis. This was somewhat surprising due to the herbaceous community, presumably with limited long-term storage. Future longer time series in different ecosystem types will enable a deeper understanding of the coupled above–below-ground action of the carbon cycle in plants. While we note our field indexes could be unstable, especially in E3, this was due to the low replication and differences between instruments rather than individual time series inconsistency. Wider application of such devices is hence reliant on reasonable per-instrument costs, the initial rationale for our instrument development.

### How close are we to true automated root phenology monitoring?

In comparison with all previous attempts to automate minirhizotron data collection and/or analysis, we produced high frequency time series in the lab and field (with support from an electrical/mechanical workshop and minimal computer science expertise). We fulfilled all objectives O1–O4 necessary for field usability. With such information, root:leaf asynchrony (Steinaker and Wilson, 2008; Steinaker *et al.*, 2010; Sloan *et al.*, 2016) can be understood from high frequency data (e.g. O4 in E1, E2, and E3). Links to C cycling (e.g. E1) such as between photosynthesis, growth, and soil/ecosystem respiration (Bahn *et al.*, 2008, 2009; Migliavacca *et al.*, 2011b) could also be understood in scalable contexts. We consider the results in the mesocosm part of this manuscript as a proof of concept and do not interpret these further, but in theory such experiments could be conducted at field scales.

The RMR workflow can process hundreds of images at the same time to annotate a single image conventionally and collecting images much more frequently than highest effort manual data collection. However, it does sacrifice some fidelity in extraction of root properties compared with manual methods. These same trade-offs have already been made in leaf phenology monitoring (e.g. using ‘greenness’ or NDVI from remote sensing rather than counting leaves or measuring leaf angles). Minirhizotron imagery contains much more relevant structural information due to the extremely local scale and, indeed, these are regularly extracted manually. There is progress in doing this automatically (Seethepalli *et al.*, 2021; Bauer *et al.*, 2022) and we expect rapid advancement in the future. Extraction using deep learning of more complex properties such as diameters and branching patterns may also be possible, although this is reliant on good quality training data and good quality images. Consistent root diameter extraction would be a good first step to achieve a calculation of ‘geometric’ root surface area (calculated from diameter and length and potentially more stable than sRSA in this study) through geometry and further conversion to biomass. However, to achieve such goals, it is important that users first pay attention to longstanding best-practice recommendation to minimize artefacts such as ensuring good contact between tube and soil, and minimizing condensation where possible (e.g. Iversen *et al.*, 2011; Rewald and Ephrath, 2013) as well as developing new image analysis techniques. Of particular additional note is superposition of roots; our analyses were also not sensitive to roots overlapping or growing close, and so our segmentation may underestimate RLD (as is generally the case in Fig. 4), but this is not a large problem in this study due to the overall low root densities. Moving from segmented images to full time series of multiple traits from multiple species in mixed communities and potentially dense root systems and tracking of individual root birth and death are ambitious and exciting goals which could be achieved by root ecologists and computer scientists collaborating in the future. Indeed, similar tasks are possible in other biological image contexts [e.g. multilabel segmentation (Kubera *et al.*, 2022), or dealing with overlapping via instance segmentation (Saleh *et al.*, 2019)]. Other CNN-minirhizotron applications can even tackle this last problem, albeit without the corrective annotation which made our approach useful for automatic data (Peters *et al.*, 2022). Other possibilities such as root colour are readily available post-segmentation but we lack a theoretical understanding of the meaning of such time series in field communities where individual species differ alongside change in time. In terms of general trends (but not absolute mass), our sRSA was also a reasonable proxy for root biomass; thus, with appropriate calibration, good quality extraction of root diameters, and density/area parameterization imagery, could be interpreted as changes in C pools and provide data to inform allocation in vegetation models. Interestingly the ‘harvest’ data point in E2 did not match the minirhizotron index, even when scaled. This is potentially explainable by the CNN, trained on live roots, outperforming humans sorting physical samples who also make mistakes in living/dead identification. In wider applications, neural networks approaches also offer the ability to limit operator bias via common scoring phases, whereas manual analysis cannot sort the same sample twice. Our datasets also contained a period of time where there was litter in the field (E3) but not a specific period of turnover which may be more difficult for CNNs, although roots did disappear from individual time series in all experiments. Because the living/dead distinction is of functional interest, further development, such as training models specifically on dead roots, using multiclassifier segmentation, or generating labels using the near-infrared or other multispectral images (Arnold *et al.*, 2017; Bodner *et al.*, 2017; Svane *et al.*, 2019) and segmenting using RGB images, may allow a segmentation of complex field imagery necessary for precise quantification of living root biomass and its complex and dynamic contribution to ecosystem C cycling.

The major advantage of the minirhizotron method is resampling over long time scales in the field (Freschet *et al.*, 2021). We were not able to make field observations over multiple years in this study and long-term reliability remains the biggest unknown. Nonetheless, we did target the most ‘difficult’ times of the year in E3 and E4 where cold and wet conditions could affect instrument performance. The accelerated clocks used in E1 and E2 also partially demonstrate long-term performance which could capture whole annual cycles if performed at similar rates in the field to those in E3. Minirhizotron studies may also use huge replication—in agricultural settings, this can reach thousands of observatories (Rajurkar *et al.*, 2022). In this case, no matter how affordable at budget an automated system is, entire coverage is unlikely to be possible with robotic systems. For this, we recommend pairing automated systems (to capture temporal dynamics) with manual systems (for spatial dynamics), ideally with exactly the same camera set-up and processing workflow. In such a design, automated phenology data can complement the most informative sampling times, and spatial understanding can inform the most representative sites for scalable temporal trends.

## Supplementary data

The following supplementary data are available at *JXB* online.

Protocol S1. Full technical instrument design.

Fig. S1. Example of sub-daily diameter artefacts found in the mesocosm.

Fig. S2. Validation of sRSA and RLD in E2, E3, and E4.

Fig. S3. Comparison between mesocosms in E2.

Table S1. Comparison of linear slope window in the E1 GAM model.

erac427_suppl_supplementary_protocol_S1_figures_S1-S3_table_S1

## Data Availability

A dataset with validation data used in this study, along with a small dataset of unannotated images, are available at Zenodo https://doi.org/10.5281/zenodo.7233828 (Nair *et al.*, 2022). The full image dataset is not sharable using current services due to its size, but the authors are happy to share this with developers of root segmentation tools on request. The code which we used to extract root length from segmented images is also available.

## Abbreviations

CNN: convolutional neural network
GCC: green chromatic coordinate
NDVI: normalized difference vegetation index
RLD: root length density
ROI: region of interest
sRSA: root surface area as measured from segmented/annotated area of images
RMR: robotic minirhizotron (the instrument developed in this study)
